# Measurement of drug concentration and bacterial contamination after diluting morphine for intrathecal administration: an experimental study

**DOI:** 10.1186/s12871-020-01151-2

**Published:** 2020-09-25

**Authors:** Aart Jan W. Teunissen, Mark V. Koning, Elisabeth J. Ruijgrok, Willem J. Liefers, Bart de Bruijn, Seppe A. Koopman

**Affiliations:** 1 Anesthesiology, Maasstadziekenhuis, Maasstadweg 21, 3079DZ Rotterdam, The Netherlands; 2 Anesthesiology, Rijnstate hospital, Arnhem, The Netherlands; 3 Pharmacy, Erasmus Medical Center, University of Rotterdam, Rotterdam, The Netherlands; 4 Pharmacy, Maasstadziekenhuis, Rotterdam, The Netherlands

**Keywords:** Morphine, Bupivacaine, Anesthesia providers, Concentration, High-pressure liquid chromatography, Dilution, Contamination

## Abstract

**Background:**

Low concentrations of morphine are required for safe dosing for intrathecal injections. Sometimes, manual dilution of morphine is performed to achieve these low concentrations, but risks dilution errors and bacterial contamination. The primary goal was to compare the concentrations of morphine and bupivacaine between four groups of syringes. The secondary goal was to investigate the difference in contamination rate between these groups.

**Methods:**

Twenty-five experienced anesthesia providers were asked to prepare a mixture of bupivacaine 2.0 mg/ml and morphine 60 μg/ml using 3 different methods as clean and precise as possible. The fourth method used was the aspiration of ampoules prepared by the pharmacy. The concentrations of morphine and bupivacaine were measured by High-Pressure Liquid Chromatography (HPLC). The medication was cultured for bacterial contamination.

**Results:**

Group 1 (median 60 μg/ml; 95% CI: 59–110 μg/ml) yielded 3 outliers above 180 μg/ml morphine concentration. Group 2 (76 μg/ml; 95% CI: 72–80 μg/ml) and 3 (69 μg/ml; 95% CI: 66–71 μg/ml) were consistently higher than the target concentration of 60 μg. The group “pharmacy” was precise and accurate (59 μg/ml; 95% CI: 59–59 μg/ml). Group 2 and “pharmacy” had one contaminated sample with a spore-forming aerobic gram-positive rod.

**Conclusion:**

Manually diluted morphine is at risk for deviating concentrations, which could lead to increased side-effects. Medication produced by the hospital pharmacy was highly accurate. Furthermore, even when precautions are undertaken, contamination of the medication is a serious risk and appeared to be unrelated to the dilution process.

## Implication statement

This study demonstrates that manual dilution of medication leads to inaccurate concentrations. This might lead to unsafe dosing.

## Background

Intrathecal administration of morphine is an effective method of analgesia. A single dose of 100–300 μg produces analgesia that lasts over 24 h [1, 2]. However, adverse events due to intrathecal morphine, such as late respiratory depression and pruritus, are described to be dose dependent [3]. The risk for adverse events may increase in doses > 500 μg [4–6]. Given this narrow therapeutic range, accurate dosing is paramount.

To achieve a safe dose of intrathecal morphine, low concentrations of morphine are necessary. However, commercially available concentrations of morphine in the Netherlands range up to 10 mg/ml or 20 mg/ml. Some health care providers use small volumes of 10 mg/ml morphine to achieve a dose of 150 μg, others dilute the morphine manually [7–9]. This leaves room for error with potentially fatal outcomes as a result.

In addition, precautions should be taken to prevent a contaminated injection, since the introduction of bacteria into the cerebrospinal fluid can lead to meningitis [10]. Even though the incidence of meningitis after an intrathecal injection is estimated to be 1:53.000, manipulations for manual dilution could contaminate the injectate and increase this incidence [11].

The objective of the current study is to measure precision and accuracy in dilution of morphine and bacterial contamination. Previous studies have investigated the dilution of morphine, but limited conclusions can be drawn due to their study design [8, 9]. The methodology was non-standardized [8] and a limited number of subjects diluted the morphine [9]. This may overestimate the accuracy of clinical practice. Moreover, bacterial contamination was not measured. In this study, various experienced anesthesia providers prepared the syringes according to three standardized methods of dilution and syringes extracted from ampoules prepared by our pharmacy. We hypothesized that the number of manoeuvres increases the risk for a dilutional error and bacterial contamination.

## Methods

For this experimental study, medical ethical approval was waived by the medical ethical committee of the.

Maasstad Hospital (Toetsingscommissie Wetenschappelijk Onderzoek Rotterdam e.o., February 5, 2018). The primary outcome was the precision and accuracy in morphine concentration within groups. The secondary outcome the difference in contamination rate between these groups.

Twenty-five experienced anesthesiologists and nurse anesthetists were asked to prepare a mixture of bupivacaine and morphine according to predefined methods. The characteristics of these methods are described in Table 1. The target concentrations were 2.0 mg/ml bupivacaine and 60 μg/ml morphine. They were provided with medication, sterilely packed syringes and needles and aseptic measures, as written below.

**Table 1:** Characteristics of dilution per method

Group	Starting concentration	Number of dilutions	Dilution volume
Method 1	10 mg/ml morphine	1	100 ml NaCl 0.9%
Method 2	10 mg/ml morphine	2	9 ml NaCl 0.9%, twice
Method 3	1 mg/ml morphine	1	9 ml NaCl 0.9%
Pharmacy	60 mcg/ml morphine 2.5 mg/ml bupivacaine	0	N/A

### Medication

For this study, commercially available medication was used for the first three methods. Specifically, bupivacaine 5 mg/ml (5 ml), morphine 1 mg/ml (1 ml) and morphine 10 mg/ml (1 ml). For the group Pharmacy, the hospital pharmacy provided ready-to-use (RTU) ampoules of 5 ml, containing a combined solution of 2.5 mg/ml bupivacaine and 60 μg/ml morphine. The ampoules were prepared under Good Manufacturing Practice (GMP) conditions by the pharmacy that is GMP certified by the Dutch Health Care Inspectorate. In short, a batch of 50 sterile ampoules were prepared. The solution was prepared in a Grade A with Grade C background aseptic cleanroom and glass ampoules were filled under nitrogen. The fluid was filtered through a 0.22 μm bacterial filter. The ampoules were sterilized in the autoclave for 15 min in 121 degrees Celsius. Quality control tests in a GMP accredited laboratory included sterility, fill volume, shelf-life, and concentration.

### Experimental design

The participants received written and oral instructions for preparation of the syringes. It was stressed that the mixtures needed to be as clean and precise as possible. The preparation of medication was performed on the Post Anesthesia Care Unit, on a clean table, specifically used for preparation of medication. As aseptic measures, caps and blue nitrile gloves, but not facemasks were worn by the participants. All ampoules were swiped with 70% ethanol before opening. The outside of the ampoules was not touched by the BD blunt fill needles (BD, Oxford, United Kingdom). After each diluting step, the providers were advised to use a new needle.

There were four methods to which a participant was obliged to prepare a syringe. Method 1 was diluting 10 mg/ml of morphine in a single step. It started with drawing up 1 ml of 10 mg/ml morphine. This was injected in a container of 100 ml NaCl 0.9%. Three ml of this mixture was aspirated into a 5 ml syringe and 2 ml of 5 mg/ml bupivacaine was added. Method 2 was a double-dilution method from 10 mg/ml of morphine. It started with drawing up 1 ml of 10 mg/ml morphine in a 10 ml syringe. This was diluted with 9 ml of NaCl 0.9% in the same syringe. After mixing, 9 ml of this content was discarded and the remaining 1 ml was diluted again with 9 ml of NaCl 0.9%. After mixing, 3 ml of this mixture was aspirated in a 5 ml syringe and 2 ml of 5 mg/ml bupivacaine was added. Method 3 was a single dilution step of 1 mg/ml morphine. It started with aspiration of 1 ml of 1 mg/ml morphine into a 10 ml syringe. This was diluted with 9 ml of NaCl 0.9%. After mixing, 3 ml of this mixture was aspirated in a 5 ml syringe and 2 ml of 5 mg/ml bupivacaine was added. For group Pharmacy the aforementioned 5 ml ready-to-use ampoule of bupivacaine and morphine was aspirated into a 5 ml syringe.

After each prepared syringe, the participants were advised to change into new nitrile gloves. Participants could perform their tasks at their own speed. Two syringes per method were prepared, leading to a total of 8 syringes per provider. Syringes were marked after each preparation. The participants started with method 1 then method 2 and proceeded to method 3. Pharmacy group contained syringes drawn from pharmacy prepared ampoules. Per method, one syringe was analysed for drug concentration and one for bacterial contamination. No materials were re-used for method 2, 3 and pharmacy group. For method 1, two samples were taken from the same 100 ml 0.9% NaCl-container.

After preparation, all the syringes were capped and analysed the same day in the pharmacy department for drug concentrations and bacterial contamination. The syringes were tested for concentration of morphine and bupivacaine by High-Pressure Liquid Chromatography (HPLC) at 285 nm in a 125 mm X 4 mm fluid column. Testing of the microbiological culture was done with the standardized method of the pharmacy department by injecting the fluid through a filter and culturing for 7 days of the filter. The contaminating microbes were identified in positive cultures.

### Statistics

A power analysis was performed with GPower 3.1. A concentration was deemed clinically acceptable if the concentration was in the range between 48 mcg/ml and 72 mcg/ml of morphine. This would be a target concentration of 80% or 120%, with an assumed standard deviation of 20%. We assumed that the concentration in the group “Pharmacy” would be a mean of 60 mcg/ml with a margin of error of 2 mcg/ml, leading to a target concentration of 100% with a deviation of 3%. In order to detect between the groups, using an alpha of 0.05 and a beta of 0.8, 10 samples per group were necessary. To increase accuracy and correct for multiple testing, 25 samples per group were obtained.

Data is described as n (%) or as median (95% Confidence Interval). The Chi-square-test was used for original data. Kruskal-Wallis was used for the testing of continuous data. A *p* < 0.005 was considered appropriate and Bonferroni correction was applied when necessary. All testing was performed with SPSS 25.0 (IBM, Armonk, New York) and graphics were made by GraphPad Prism version 7.1 (GraphPad Software, San Diego, California).

## Results

**Fig. 1:**
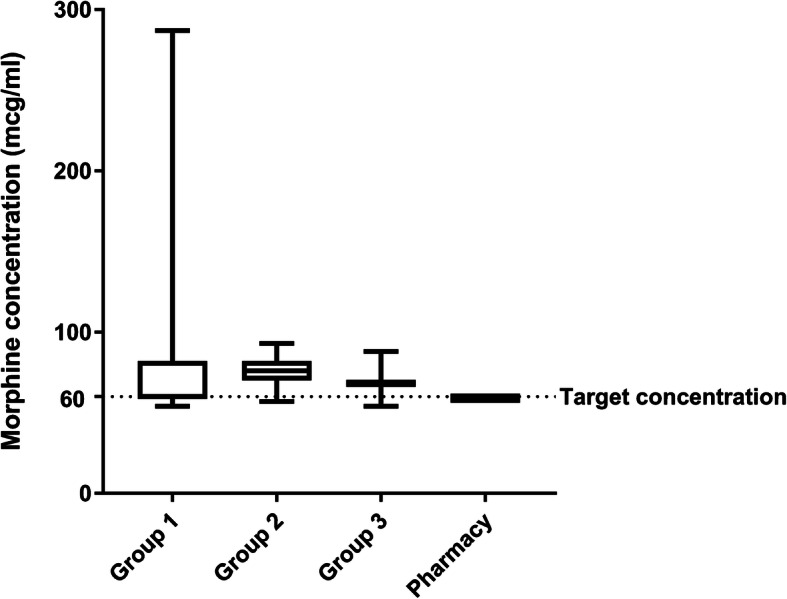
Morphine concentrations. Box and whiskers demonstrate the median, interquartile range and range

All continuous data showed a non-normal distribution (Shapiro-Wilk-test, *p* < 0.05). The distribution of the morphine is displayed in Fig. 1. Details of morphine and bupivacaine concentrations are presented in Table 2. Group 1 had three outliers with morphine concentrations of 189, 246 and 287 μg/ml. Morphine concentrations were most precise in the pharmacy group (59 μg/ml; 95% CI of 59–59 μg/ml) followed by group 3 (69 μg/ml; 95% CI of 66–71 μg), group 2 (76 μg/ml; 95% CI of 72–80 μg/ml) and finally group 1 (60 μg/ml; 95% CI of 59–110 μg/ml). Groups 2 and 3 reached higher concentrations than the pharmacy group (*p* = 0.000, Fig. 1).

**Table 2:** Details of concentrations of morphine and bupivacaine

Group	Method 1	Method 2	Method 3	Pharmacy
**Morphine concentration (μg/ml)**	60 (59–110)	76 (72–80)	69 (66–71)	59 (59–59)
**Bupivacaine (mg/ml)**	1.98 (1.73–2.06)	2.00 (1.95–2.03)	1.97 (1.91–2.00)	2.54 (2.54–2.55)
**Morphine Out-of-range (< 80%)**	0 (0%)	0 (0%)	0 (0%)	0 (0%)
**Morphine Out-of-range (> 120%)**	7 (28%)	18 (72%)	3 (12%)	0 (0%)
**Bupivacaine** **Out-of-Range (< 80%)**	1 (4%)	0 (0%)	1 (4%)	0 (0%)
**Bupivacaine** **Out-of-Range (> 120%)**	0 (0%)	1 (4%)	0 (0%)	0 (0%)

Data presented as median (95% CI) or n (%) where appropriate

Accuracy was calculated as the difference between the individual measurements and the target-concentration. No difference in accuracy between group 2 and 3 was detected (16 μg (10–22) [2–33] vs. 9 μg (6–15) [1–28], *p* = 0.329). The pharmacy group was the most accurate (*p* = 0.000 for all comparisons with groups 1, 2 and 3). No difference in accuracy between groups 1 and 3 was detected (2 μg (95% CI: 2–51) vs. 9 (95% CI: 7–11 μg), *p* = 0.645).

Bupivacaine concentrations were not different between groups 1–3 (*p* = 1.000 for all comparisons). Since the pharmacy group had a higher concentration, we calculated the difference between the target and the measured concentrations. Again, no difference was detected (*p* = 1.000 for all comparisons).

There was no relation detected between the anesthesia provider and the accuracy of the morphine concentrations (*P* = 0.462).

Two samples had positive cultures with spore-forming aerobic gram-positive rods (group 2 & 4) (*p* = 1.000). These were prepared by the same provider. In one sample a fiber was detected (group 2).

## Discussion

Our results show that diluting a medicine manually is prone to deviating concentrations, even by experienced personnel. The concentrations in the group “Pharmacy” were precise and accurate. Of the manual dilution methods, the 3rd led to most accurate and precise concentrations. This means that a lower starting concentration leads to higher precision and accuracy. Contamination occurred in groups 2 and pharmacy group.

The dilution method in group 1 resulted in three cases with concentrations which could result in respiratory depression when injected intrathecally. If five millilitres of these solutions would have been injected it would result in an injected dose of 1 to 1.5 mg intrathecally, while the targeted dose is 300 μg. Therefore, this method should not be used. It illustrates that this dilution process is at risk of creating dangerously high morphine concentrations. The erroneous high concentration is possibly achieved because no new extraction needle was used and by the lack of rinsing off the first extracting needle in this protocol, thereby leaving a small volume of high concentration morphine in the needle. A second explanation may be because the solution did not mix properly in the 100 ml container [9].

The precision of group 2 and 3 was clinically acceptable, even though the accuracy was limited. When these methods of dilution are clinically applied, one has to aim for the lower limit of the therapeutic range of intrathecal morphine to prevent an inadvertently high dose. The higher concentration is possibly caused by the excess volume of a 1.0 ml morphine ampoule, which has to be more than 1.0 ml to allow an extractable volume of 1.0 ml [12]. We believe that methods 2 and 3 are inherently safer methods, because the needle is rinsed if no new needle is used and the solution is mixed by aspiration of 9 ml of saline. This is supported by the study of Benkhadra et al., which shows that mixing of the syringe results in a homogenous distribution of the solution [9]. Even though this was a relatively minor effect, every cause for imprecision should be excluded.

Most remarkably, 2 groups contained a contaminated sample, despite precautions of clean preparation, such as wearing non-sterile gloves and caps and swiping the ampoule with 70% ethanol before opening. In addition, this study was performed in the Post Anesthesia Care Unit, a room where intrathecal injections are commonly performed. We did not instruct the participants to wear face masks, because we prepared the solutions as in daily practice. Dilution steps did not appear to increase bacterial contamination. Given the rate of contamination, it is surprising that the incidence of bacterial meningitis after an intrathecal injection is around 1: 53.000 [11].

Several pathways for contamination of intrathecal injection are described. One pathway consists of bacteria originating from the oropharynx of the healthcare provider falling on the sterile area and instruments. The aerobic spores are predominantly found on skin and materials, but seldom in the human oral cavity, making it unlikely that wearing a face mask during preparation would change the results. A second pathway is that contaminated particles fall in the ampoule when this is opened [10, 13]. Based on the identified spores, this pathway is more likely. The current study shows contaminated medication could be an important pathway of introducing a microorganism into the cerebrospinal fluid and our precautions fail to prevent contamination by this bacterium [14].

This contamination with aerobe gram-positive rods is most likely *Bacillus cereus* which is also part of the human skin flora and commonly associated with contamination [15]. The spores of *Bacillus cereus* are alcohol-resistant [16]. In healthy patients, the possibility of a *Bacillus cereus* infection in the central nervous system is low because of intact host resistance. In immunocompromised patients, however, *Bacillus cereus* was identified as causative microorganism for meningitis leading to fatal outcomes [17, 18]. The inability to remove the spores with alcohol might pose a risk in immunocompromised patients, even though a *Bacillus cereus*-meningitis is rare. It is possible although difficult to remove the spores of *Bacillus cereus* by using disinfection procedures with solutions containing high concentration chlorine or hydrogen peroxide.

Bupivacaine was added to measure control of volume. This study showed that the aspiration of 2 ml into a 5 ml syringe is accurate. Furthermore, bupivacaine has antibacterial properties, making it of interest for the measurement of contamination [19]. Despite this antibacterial effect, contamination occurred in 2% of the syringes. The difference in bupivacaine concentration between group pharmacy (2.5 mg/ml) and the other groups (2.0 mg/ml) is unlikely to affect the contamination rate [20, 21].

A few recommendations can be made based on this study. Firstly, prefabricated drugs should be preferred in clinical practice. In some countries, dilution of medication is regarded as compounding of medication and is subject to strict regulations. If prefabricated medication is not available, one should dilute from the lowest possible starting concentration and mix the syringe during the process.

Secondly, sterile precautions should be undertaken when medication for intrathecal use is prepared, since bacterial contamination is likely to occur as shown by Zacher et al. [13] Several hospitals routinely prepare drugs with high microbiological risk, such as intrathecal administrations, in a cleanroom environment, either centrally in the hospital pharmacy or decentral in a laminar flow cabinet in close proximity to the operation theatres. Contamination occurs during dilution or aspiration of medication. Our study fails to demonstrate a lower risk of contamination with ready to use (RTU) medication. Prefilled sterile syringes, which are ready to administer (RTA) could be an alternative for (RTU) medication. Use of these prefilled syringes would avoid drawing up the medication. The effects of using prefilled sterile syringes on contamination should be investigated further.

Thirdly, clinical studies regarding intrathecal morphine sometimes do not describe the manufacturing process [22] or dilute manually [23]. Manually diluted drugs could yield a variance in dose with a different effect/side effect ratio. Therefore, the manufacturing process should be described in clinical studies.

A limitation of this study is that we did not determine the species beyond the gram-stain.

Gram positive aerobe spore forming bacteria are either Bacillus antracis or *Bacillus cereus*. The first would be very unlikely. Additionally, the bupivacaine concentration in the group pharmacy differed from the other groups, although this range of bupivacaine is unlikely to affect the contamination rate of *Bacillus cereus* [21]. Also, the incidence of contamination was not less in the pharmacy group.

## Conclusion

Manual dilution of medication may lead to inaccurate concentrations. We recommend using prepared solutions from the hospital pharmacy. If these are not available, it is advised to use the lowest starting concentration available. In studies, the medication should be produced by the pharmacy since manual dilution can cause an erroneous dose. Contamination of medication is a serious risk and precautions should be taken seriously, even though in this study it appeared to be unrelated to the method used.

## Data Availability

The datasets used and/or analysed during the current study are available from the corresponding author on reasonable request.

## Abbreviations

mg: Milligram
μg: Microgram
ml: Millilitre
μm: Micrometre
nm: Nanometre
HPLC: High-Pressure Liquid Chromatography
CI: Confidence interval
GMP: Good Manufacturing Practice
BD: Becton Dickinson
RTA: Ready to administer
RTU: Ready to use
